# Two-Dimensional Quantum Dot-Based Electrochemical Biosensors

**DOI:** 10.3390/bios12040254

**Published:** 2022-04-17

**Authors:** Jian Zhang, Xiaoyue Zhang, Sai Bi

**Affiliations:** College of Chemistry and Chemical Engineering, Qingdao University, Qingdao 266071, China; 2019020387@qdu.edu.cn (J.Z.); 2020020337@qdu.edu.cn (X.Z.)

**Keywords:** two-dimensional quantum dots (2D-QDs), synthesis methods, electrochemical biosensors, DNA sensors, immunological sensors, enzyme sensors, aptasensors

## Abstract

Two-dimensional quantum dots (2D-QDs) derived from two-dimensional sheets have received increasing interest owing to their unique properties, such as large specific surface areas, abundant active sites, good aqueous dispersibility, excellent electrical property, easy functionalization, and so on. A variety of 2D-QDs have been developed based on different materials including graphene, black phosphorus, nitrides, transition metal dichalcogenides, transition metal oxides, and MXenes. These 2D-QDs share some common features due to the quantum confinement effects and they also possess unique properties owing to their structural differences. In this review, we discuss the categories, properties, and synthetic routes of these 2D-QDs and emphasize their applications in electrochemical biosensors. We deeply hope that this review not only stimulates more interest in 2D-QDs, but also promotes further development and applications of 2D-QDs in various research fields.

## 1. Introduction

Graphene has greatly influenced many fields since its inception in 2004, and its success has also sparked the enthusiasm of researchers to explore other two-dimensional (2D) layered inorganic nanomaterials. Over time, more 2D materials have been discovered, such as hexagonal boron nitride (h-BN) [1,2], black phosphorus (BP) [3,4], graphitic carbon nitride (g-C_3_N_4_) [5,6,7], MXene [8,9], transition metal dichalcogenides (TMDCs) [10,11], and transition metal oxides (TMOs) [12,13], to meet the new application requirements. In their thick bulk form, the atoms in each layer are firmly linked together by covalent bonds, coordinate covalent bonds or ionic bonds, and the layers are connected by relatively weak van der Waals forces. When the lateral dimension of these 2D materials is reduced below 100 nm (typically < 10 nm), the 2D quantum dots (2D-QDs) can be generated due to the strong quantum confinement effect [14,15]. Thus, the 2D-QDs generally refers to quantum dots derived from 2D materials [15].

Sometimes these 2D-QDs are considered zero-dimensional (0D) materials, but in essence, the 2D-QDs are just smaller forms of 2D-layered materials and still maintain their two-dimensional lattices. Compared with the original forms, the 2D-QDs have better solubility and dispersion, and a larger surface volume ratio, which are more easily doped and functionalized with retaining the original advantages of low toxicity, chemical inertia, and excellent electronic properties. Diverse properties and properties enable the 2D-QDs to be readily applied in various fields, including catalysis, energy storage, and optoelectronics to sensing, bioimaging, and cancer therapy [16,17,18,19].

**Figure 1:**
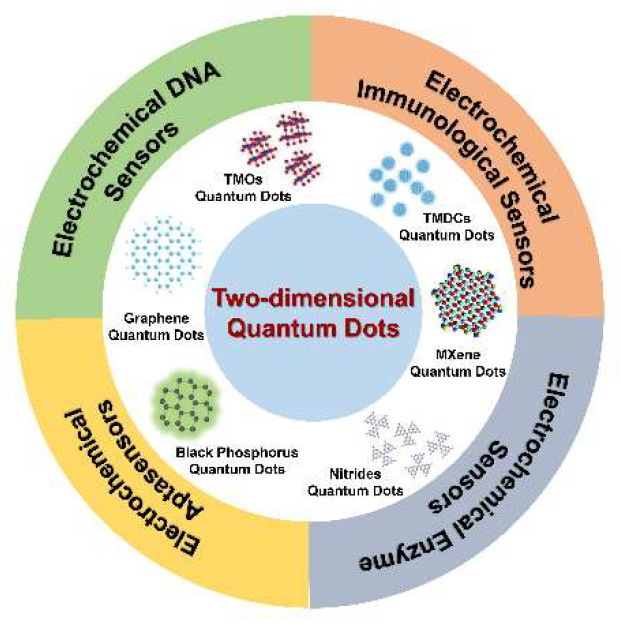
Schematic diagram of 2D-QD-based electrochemical biosensors.

From disease diagnosis to detecting biological agents in the environment, biosensors are closely relevant to our modern life. Among various biosensors, the electrochemical biosensors have demonstrated the advantages of high sensitivity, high signal-to-noise ratio, relative simplicity, and fast response times in the detection of target analytes. The electrochemical biosensors combine the analytical capabilities of electrochemical techniques with the specificity of recognition processes. For the basic principle of electrochemical biosensors, the biomolecules immobilized on electrodes chemically react with analytes to generate or consume ions or electrons, resulting in changes in electrical signals, including potential, current, and impedance. Based on the different molecular recognition elements, the electrochemical biosensors can be mainly classified into electrochemical DNA sensors, electrochemical immunological sensors, electrochemical enzyme sensors, electrochemical aptasensor, and so on. Moreover, the development of nanomaterials provides a wide range of candidates to improve the stability, selectivity, and sensitivity of electrochemical sensors. The versatile nanomaterials can synergistically enhance the catalytic activity, signal transduction, and selectivity of electrochemical biosensors. In particular, the emergence of 2D-nanomaterials provides new opportunities to improve the performance of biosensors. Compared to other nanomaterials, two-dimensional nanomaterials have large specific surface area and excellent optical/electrical properties, making them the preferred choice for sensor design. More importantly, due to the excellent electrical conductivity and high current response sensitivity, 2D-QDs have greatly promoted the development of electrochemical biosensors (Figure 1) [20,21,22,23,24]. Although different 2D-QDs vary in the prominent properties, they share some common features that make them promising for constructing electrochemical biosensors: (1) electrochemical activity; (2) electrical conductivity; (3) large surface-to-volume ratio; (4) ease of functionalization. Due to these properties, the utilization of 2D-QDs in electroanalysis can greatly improve the detection performance.

During the past decade, although the 2D-QD-based electrochemical sensors have received extensive attention and research, there are only a few reviews focusing on the application of 2D-QDs in electrochemical biosensors. In this review, we introduced the category and synthesis of 2D-QDs and summarized the recent advances of 2D-QD-based electrochemical sensors for the detection of biomolecules in the last five years, hoping to provide new insights and ideas for the design and construction of electrochemical devices. In addition, we also point out the challenges and future perspectives related to the development of 2D-QD-based electrochemical biosensors.

## 2. Categories of 2D-QDs

In the past decade, thanks to the rapid development of two-dimensional materials, a large number of 2D-QDs have been synthesized. In theory, all QDs could be generated from their native bulk-layered forms. Compared with the original 2D form, 2D-QDs are not only reduced versions of them, but they also exhibit some new characteristics and properties due to the quantum confinement effect and edge effect. In this section, we discuss the existing 2D-QDs based on different 2D materials, including graphene, black phosphorus, nitrides, TMDCs, TMOs, and MXenes, and their unique properties.

### 2.1. 2D Graphene QDs

Graphene is a honeycomb-shaped 2D material composed of a single layer of carbon atoms. The highly ordered and closely packed structure of graphene determines its unique properties [25]. One of these properties is the zero-energy bandgap, which arises from the infinite physical dimension and defect-free crystal structure of graphene. This property highly impedes the application of graphene in optoelectronic and electronic fields. Therefore, by limiting the size and introducing defects, the bandgap of graphene can be tuned from 0 eV to higher states, eventually forming non-zero bandgap materials, such as graphene QDs (GQDs) [26]. Generally, the thickness of GQDs is less than 10 nm and the transverse dimension is less than 100 nm. In fact, GQDs are considered to be size-reduced graphene sheets. However, due to the significant change in physical size, GQDs show more advanced properties compared to graphene, such as larger specific surface areas, greater surface active sites, and more available edges [27,28]. Owing to the quantum confinement and edge effects, GQDs exhibit faster electron transport and higher conductivity, making them high-performance electron transporters. In addition, the larger specific surface area enlarges the contact of GQDs with analytes, which facilitates the interaction between GQDs and electroactive species, and also promotes the direct electron transfer (DET) from enzymes and proteins. Therefore, the introduction of GQDs has significant effects, i.e., in improving the electrochemical reaction rate. Moreover, the functional groups contained in GQDs allow them not only to obtain excellent solubility, but more importantly, to get more powerful capabilities to functionalize with organic, inorganic, or biological moieties, which is crucial for the design of electrochemical biosensors [20,26,27,28,29,30].

### 2.2. Nitride-Based 2D-QDs

Graphitic carbon nitride and h-BN-based QDs (BNQDs) are the two most popular nitride-based QDs. Theoretical calculation results demonstrate that the graphitic carbon nitride exists in five forms, α-C_3_N_4_, β-C_3_N_4_, g-C_3_N_4_, cubic C_3_N_4_ and pseudo-cubic-C_3_N_4_, in which the g-C_3_N_4_ is the most stable allotrope among them [31]. The structure of g-C_3_N_4_ is similar to that of graphene, which can be regarded as N heteroatoms, substitute C atoms in the graphene skeleton with the formation of a π-conjugated system containing sp^2^ hybridized N and C atoms. Although g-C_3_N_4_ sheets and graphene sheets share the similar two-dimensional structure, their properties are significantly different (and so as to their QDs). For example, the photoluminescence quantum yield of g-C_3_N_4_ QDs is higher than that of GQDs, making them promising candidates as biosensors [32,33]. In BNQDs, boron and nitrogen atoms are alternately linked by covalent bonds to form a hexagonal structure similar to GQDs, so BNQDs are also called “white graphene QDs” [34]. BNQDs have fascinating physical and chemical properties, such as high thermal conductivity, exceptional chemical stability, low toxicity, excellent biocompatibility, and selectivity [35]. In particular, the electronic, magnetic, and optical properties of h-BN QDs are highly tunable.

### 2.3. Black Phosphorus 2D-QDs

Phosphorene is a black phosphorus (BP) crystal with an atomically-thin layer, in which each phosphorus (P) atom covalently binds with three other atoms to form a folded honeycomb structure. In 2015, Zhang and coworkers first reported the preparation of BPQDs from bulk black phosphorus crystals using a facile top-down method in a solution phase [36]. Compared to traditional 2D BP nanosheets, the emerging BPQDs exhibit some unique properties and demonstrate great potential for a broad range of research fields, including sensing, catalysis, biotechnology, and biomedicine [37]. Phosphorene and its QDs are sensitive to both water and air, which is a hindrance to the synthesis, characterization, and application of BPQDs [38,39,40]. However, it can be used in energy devices, such as lithium-ion batteries or sodium-ion batteries that work in anhydrous and oxygen-free conditions. Interestingly, oxidized BPQDs have better water solubility and near-infrared absorption, which can be exploited for photothermal therapy [41,42].

### 2.4. TMDCs-Based 2D-QDs

TMDCs are a fascinating family of 2D materials with an X-M-X sandwich structure, where M, representing metal ions, is generally Ti, Zr, V, Nb, Mo, W, Hf, Ta, and X, representing chalcogen atoms, is usually S, Se, or Te, respectively. At the atomic level, the chalcogen atoms and metal atoms within the same layer are stably held together by covalent bonds. However, the different layers connect each other only by weak van der Waals forces [43]. The structures and properties of these TMDCs QDs are quite stable, and their electron mobility is even comparable to that of silicon, allowing them to be used to fabricate transistors. In contrast with TMDCs, TMDC QDs exhibit many fresh characteristics, including higher bandgap, a large surface to volume ratio, abundant active sites, good electrical conductivity, and fast heterogeneous electron transfer. These excellent advantages have made TMDC-based 2D QDs potential candidates for future electrochemical sensor devices [44,45].

### 2.5. TMO-Based 2D-QDs

TMO-based 2D-QDs, where M is usually Ti, Zr, V, Nb, Mo, and W, are different from TMO-based 2D-QDs because of their relatively smaller band gaps. Here, the most investigated TMO-based 2D-QDs are MoO_x_ (x < 3) and WO_3−x_, both of which show semiconductor characteristics. Generally, the synthesis of MoO_x_ QDs is achieved with H_2_O_2_ and MoS_2_ as precursors. In addition, the MoO_x_ QDs have excellent photoluminescence properties, so they can be used as photoluminescence probes in chemical sensing [46,47,48]. The reported WO_3−x_ QDs mainly exist in the form of WO_3_ and W_18_O_49_. WO_3−x_ QDs have excellent optical and thermal stability and electrical properties, which can be used for sensing, bio-imaging, solar cells, electromagnetic wave absorption, and other applications [49].

### 2.6. MXenes-Based 2D-QDs

MXenes, a large family of two-dimensional transition metal carbides, carbonitrides, and nitrides, have attracted great interest in sensing owing to their structural similarity to graphene. MXenes are formed by selective etching A-element from their MAX phases with the chemical formula M_n+1_X_n_ (n = 1, 2, or 3), where M represents the early transition metals of Mo, Ti, Sc, Zr, V, Hf, Ta, Nb, Cr, etc., A is mainly III-A and IV-A group elements, and X is usually the carbon and/or nitrogen element. This special structure enables MXenes to possess metal-like properties, namely high electrical and thermal conductivity. Regarding their 2D-QDs, the ultra-thin size corresponds to a large specific surface area and high density of functional groups, which enables higher density to bind biomolecules, thereby enhancing the performance of biosensor [24].

## 3. Synthetic Methods of 2D-QDs

In general, the synthesis methods of 2D-QDs can be divided into two broad categories: top-down approaches, which synthesize 2D-QDs by cleavage of relatively large bulk precursors (Table 1), and bottom-up approaches, which synthesize 2D-QDs by aggregation and growth of small organic or inorganic molecules (Table 2).

### 3.1. Top-Down Methods

#### 3.1.1. Ultrasonication-Assisted Methods

Sonication is one of the most versatile methods for 2D-QDs synthesis, and most layered materials can be transformed into 2D quantum dots by this method. Briefly, the bulk raw material is sonicated in the appropriate solvent, and sometimes the high temperature or high pressure is also required. In this method, the sonication induces liquid cavitation and generates bubbles in the solution. Moreover, the collapse of bubbles will cause the violent tensile stress on the surface of layered bulk materials to destroy the bonds, realizing the exfoliation of these crystals and finally forming the 2D-QDs [70]. Most 2D-QDs can be obtained by this method, such as GQDs [50], BPQDs [36,51], g-C_3_N_4_ QDs[52,53], BNQDs [54], and MoS_2_ QDs [55]. Despite the advantages of simplicity and ease of operation, the ultrasound-assisted methods are usually carried out in organic phases, which limit the application of QDs in aqueous solutions. Therefore, several methods to fabricate 2D-QDs in aqueous environments have been developed over the years. For instance, using natural graphite, expanded graphite, and graphite oxide as raw materials, Gao and coworkers synthesized three kinds of GQDs: pristine graphene quantum dots (PGQDs), expanded graphene quantum dots (EGQDs), and graphene oxide quantum dots (GOQDs), in an ultrasound-assisted supercritical CO_2_/H_2_O system [50]. Both the intense knocking force generated from the high-pressure acoustic cavitation and the superior penetration ability of scCO_2_ are the keys to obtaining these products. In addition, Lee and coworkers obtained a new class of fluorescent BPQDs from black phosphorus using an exfoliated solution method with sonication in an aqueous phase [51]. The BPQDs were able to keep stable for 10 days in an aqueous solution and exhibited excitation wavelength-dependent photoluminescence characteristics, which have great potential for biomedical applications.

#### 3.1.2. Hydro/Solvothermal Methods

Hydrothermal and solvothermal methods are the most practical techniques with great flexibility and controllability, which have been widely used in the synthesis of various QDs in the past decade [56,57,58]. In brief, after the precursor is dispersed in the solvent, the operation is carried out in a closed container and reacts at a high temperature. The principle of this method is to destroy the bonds by thermal shear force under high temperature and high pressure to exfoliate the layered 2D flakes into ultrasmall QDs. Importantly, the characteristics and properties of solvothermally synthesized 2D-QDs are closely related to the reaction temperature, solution/reactor volume ratio, processing time, concentration, solvent type, etc. For example, Xue et al. developed a size-tunable monolayered Ti_3_C_2_ MXene QDs through the facile hydrothermal method [59]. By adjusting the reaction temperatures, the MXene QDs with different sizes and atomic concentrations of Ti can be obtained. Furthermore, the hydrothermal and solvothermal methods can be used to synthesize heteroatom-doped 2D-QDs. Xu et al. produced an MXene QD with high photoluminescence quantum yield using Ti_3_C_2_ as a precursor and ethylenediamine as a nitrogen source by the hydrothermal method [57]. The as-synthesized Ti_3_C_2_ QDs can be applied for highly sensitive detection of heavy metal ions (Fe^3+^) and hydrogen peroxide, which demonstrate broad prospects in the field of biosensing.

#### 3.1.3. Ion Intercalation-Assisted Methods

As a typical top-down approach, the ion intercalation-assisted strategy is widely used to cleavage layered bulk 2D materials into 2D-QDs. The basic principle is to intercalate cations into the interlayer gaps, thereby increasing the interspace and weakening the van der Waals forces. Thus, these layered materials can be easily exfoliated into single or multiple layers. The unique mechanism has sparked enthusiasm to explore the synthesis of 2D-QDs by this method, such as MoS_2_ QDs [61,62], BNQDs [63], and MXenes QDs [71]. For the preparation process, the bulk raw material is first immersed in a solution containing metal cations for the intercalation of ion, and the exfoliation is then performed using mechanical force. This strategy has the significant advantages in the preparation of single-layer 2D-QDs, but the production yield is relatively low. Moreover, the process is relatively complicated and the obtained product requires additional purification to remove the introduced cations. For instance, Qiao and coworkers demonstrated an effective multi-exfoliation method to prepare monolayer MoS_2_ QDs from MoS_2_ powder via lithium intercalation [61]. Pristine MoS_2_ powder was immerged in n-butyl lithium solution to obtain Li_x_MoS_2_ for the first intercalation, and the exfoliation was achieved by ultrasonicating Li_x_MoS_2_ in water. The intermediate product was then subjected to the same two-step procedure twice to obtain the final MoS_2_ QDs. The as-synthesized QDs were monolayer with a lateral size around 3 nm. In addition, Zhu et al. proposed a method to prepare single-layered Ti_3_C_2_ QDs from multilayered MXenes by ion-intercalation in aqueous tetramethylammonium hydroxide (TMAOH) [71]. The reaction with TMAOH induced both intralayer cutting and interlayer delamination, which facilitated the conversion into small pieces and monolayers. The resulting product exhibited bright and tunable fluorescence with a monolayer thickness of 1 nm, while the original chemical structure was well preserved. Moreover, this method is also applicable to the synthesis of other MXene 2D-QDs.

#### 3.1.4. Microwave-Assisted Methods

Microwave-assisted methods are one of the most efficient and economical processes used for synthesizing 2D-QDs, in which the heat generated by microwave can weaken the bonds and interlayer van der Waals forces. This method can greatly reduce the time to synthesize the 2D-QDs, which can be obtained in a few minutes. More importantly, the QDs synthesized by the microwave-assisted method have relatively high quantum yields [64,65]. For example, through a microwave-assisted pathway, Yin and coworkers synthesized the g-C_3_N_4_ QDs with high crystallinity by direct transformation of bulk g-C_3_N_4_ [66]. In a typical run, the g-C_3_N_4_ powder was added to the alumina crucible and a beaker was placed upside down on the crucible. The whole apparatus was then microwaved in a household microwave oven for 5 min. Finally, the sediment on the inner wall of the beaker was collected and dispersed with ethanol, followed by removing the large particles by centrifugation. The collected products with an average diameter of 3.5 ± 0.5 nm exhibited excellent photoluminescence characteristics with a quantum yield of ∼17%.

### 3.2. Bottom-Up Methods

The most significant difference between top-down and bottom-up approaches is the precursor difference. In contrast to the bulk crystals used in the top-down approach, the bottom-up approach typically involves the fusion of small precursor molecules into larger structures to form QDs, which has demonstrated the significant advantages in surface modification and particle size distribution control of quantum dots. The hydrothermal/solvothermal method is the most common one for bottom-up synthesis of QDs. During the hydrothermal/solvothermal synthesis, the high temperature and pressure conditions are used to crystallize precursors into the desired material in the solvent, which is relatively easy to introduce heteroatom doping. For example, Ganganboina et al. synthesized a nitrogen atom-doped GQDs (N-GQDs) using citric acid and urea as raw materials, in which urea was used as the nitrogen source [66]. The highly fluorescent N-GQDs with a quantum yield of 0.34 were then deposited onto V_2_O_5_ nanosheets for highly selective and sensitive fluorescence detection of cysteine. In another study, nitrogen and sulfur doped GQDs (N, S-GQDs) were synthesized by adjusting the mass ratio of thiourea:citric acid to 1:1 [67]. Equal masses of citric acid and thiourea were dissolved in deionized water with stirring, and the solution was then continuously heated in the autoclave to obtain the final product. The average lateral size of the as-prepared N, S-GQDs was 4.8 ± 0.5 nm and the lattice fringes were 0.21 nm, which was consistent with the (100) plane of GQDs, demonstrating the successful synthesis of N, S-GQDs. The external microwave and laser assisted for synthesizing quantum dots also have broad applications in top-down pathways and various 2D-QDs have been fabricated by this way, such as GQDs, g-C_3_N_4_ QDs, and BNQDs. This approach relies on the dense and uniform energy provided by microwaves and lasers to accelerate the chemical reactions that convert precursors into 2D-QDs. For example, using guanidine hydrochloride and EDTA as the precursors, Tang et al. prepared g-C_3_N_4_ quantum dots with strong fluorescence and high quantum yield under the assistance of microwave [68]. The as-prepared g-C_3_N_4_ QDs were able to exhibit the chemiluminescence ability in the presence of NaClO, widely expanding the application of g-C_3_N_4_ QDs in chemiluminescence and providing a new insight into the optical characteristics of the g-C_3_N_4_ QDs. In addition, Liu et al. synthesized two sulfur-regulated BNQDs (S-BN QDs^T^ and S-BN QDs^L^) by the microwave-assisted hydrothermal method using boric acid and melamine as boron and nitrogen sources, and thiourea and L-cysteine as two different sulfur sources [69]. The average diameter of S-BN QDs^T^ (9.8 nm) was slightly larger than that of S-BN QDs^L^ (9.2 nm), and the corresponding lattice fringes were 0.76 nm and 0.72 nm, respectively. Moreover, since the functional groups introduced by thiourea and L-cysteine on the surface of BN QDs were different, the electrooptic properties of the two QDs were also different from each other. The ECL intensities of S-BN QDs^T^ and S-BN QDs^L^ were increased by 1.67 and 2.59 times compared with BN QDs, respectively.

However, most bottom-up methods are only applicable to the synthesis of a few 2D-QDs and lack universality. Moreover, most of these methods are based on the wet synthesis and the experimental parameters, such as the precursor concentration, reaction temperature, solvent system, and surfactant used, have a great influence on the morphology and properties of 2D-QDs. Therefore, many bottom-up approaches require further development compared to top-down synthetic routes.

## 4. Applications of 2D-QDs in Electrochemical Biosensors

In recent years, 2D-QDs have been widely applied in the construction of electrochemical DNA biosensors, benefiting from their advantages of easy immobilization of biomolecules, good biocompatibility, and high multifunctionality. In this section, we will focus on recent research on the 2D-QD-based electrochemical biosensors, including DNA sensors, immunological sensors, enzyme sensors, and aptasensors (Table 3).

### 4.1. 2D-QD-Based Electrochemical DNA Sensors

**Figure 2:**
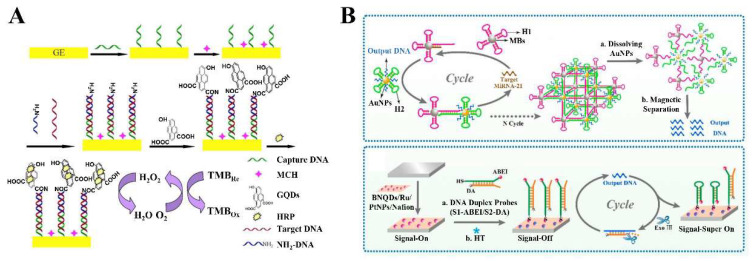
The 2D-QD-based electrochemical DNA biosensors. (**A**) Detection of microRNA-155 using GQD-based electrochemical biosensor. Adapted with permission from Ref. [72]. Copyright 2016 Elsevier. (**B**) Construction of ECL biosensor based on BNQDs/Ru/PtNPs/Nafion nanocomposites combined with 3D DNA network for signal amplification. Adapted with permission from Ref. [74]. Copyright 2019 American Chemical Society.

The detection of DNA/RNA with high specificity and sensitivity is of great significance in the fields of biochemistry and biomedicine, such as gene profiling, drug diffusion, and clinical diagnostics [101,102]. Over the past decade, a wide variety of DNA sensing methods and techniques have been developed, among which electrochemical sensing is particularly popular because of its advantages of high sensitivity, strong specificity, low limits of detection (LOD), and wide linear range [103]. In addition, the emergence of functionalized nanomaterials further drives the development of electrochemical biosensors [104]. The combination of electrochemical biosensors with conductive micron/nanoscale materials will increase the sensitivity and selectivity of sensors. In this context, the 2D-QDs hold great potential in electrochemical sensing due to their unique characteristics, such as high surface activity, strong adsorption capacity, improved electron transfer efficiency, easy functionalization, and immobilization of biomolecules. For instance, Hu et al. designed a GQD-based electrochemical biosensor for sensitive detection of microRNA-155 (Figure 2A) [72]. The abundant carboxyl groups at the edge of GQDs were linked to the NH_2_-DNA on the electrode through amide bonds, and served as the carriers for immobilizing horseradish peroxidase (HRP). Due to the catalysis of GQDs and enzyme, the GQD-based electrochemical biosensor obtained a good linear range (1 fM–100 pM) and low LOD (0.14 fM) in the determination of microRNA-155. Based on a GQD-modified pencil graphite electrode (GQDs/PGE), Akbarnia and coworkers reported a label-free DNA assay for the detection of microRNA-541 [73]. To fabricate the biosensor, GQDs were electrodeposited on the surface of a bare PGE, and the probe was immobilized on the GQDs/PGE through the reaction between –NH_2_ of the probe and –COOH of GQDs. Then, the probe was hybridized with microRNA-541 and treated with Hinf1. The electrochemical response of the genosensor was achieved through monitoring the intrinsic electroactivity of guanine in the remaining dsDNA. The presence of GQDs favored the oxidation of guanine and adenine due to their high mobility for charge carriers. The proposed method can distinguish the microRNA-541 sequence from the non-complementary sequence with the single-base mismatch at recognition sites. In addition, a voltammetry method based on amino functionalized GQDs (NH_2_-GQDs) was constructed to detect microRNA-25 [22]. In this work, the NH_2_-GQDs were used to modify glassy carbon electrodes (GCE) to generate amplified electrochemical signals and provide a large active site to capture amino-linked DNA probes on the electrode surface. The results showed that the electrochemical biosensor can well discriminate the microRNA-25 with other non-target microRNAs even with single-base mismatch. The proposed GQD-based electrochemical biosensor offered a linear range from 0.3 nM to 1.0 μM with a LOD of 95.0 pM for microRNA-25.

Recently, the researchers have found that the synergistic effect of the 2D-QDs and other nanomaterials can further improve the performance of electrochemical biosensors. For example, Ounnunkad’s group constructed an AuNPs/GQDs/GO nanocomposites modified three-screen-printed carbon electrode (3SPCE) array for simultaneous detection of three microRNAs [21]. Benefiting from the synergetic effect of AuNPs, GQDs, and GO, the highest oxidation peak current and the lowest peak-to-peak separation were observed from the AuNPs/GQDs/GO nanocomposite-modified 3SPCE surface, resulting in the LODs as low as fM level. In addition, the 2D-QDs have also played an important role in the design and improvement of electrochemiluminescence (ECL)-based sensors. However, the long electron transfer distance between the 2D-QDs in the solution and luminophore on the electrodes often reduce the ECL efficiency. To overcome this issue, Zhang et al. prepared a nanocomposite (BNQDs/Ru/PtNPs/Nafion) by dispersing Ru(bpy)_3_^2+^ and BNQDs in Nafion solution containing Pt nanoparticles (Figure 2B) [74]. Because of the shortened electron transfer distance between BNQDs and Ru(bpy)_3_^2+^, a strong initial ECL response was achieved. In addition, the synergistic effect of 2D-QDs and nanoparticles also induces the surface plasmon oscillations when the electrochemically induced excited-state luminescence is coupled to the noble metal surface, which can effectively enhance the ECL signal of QDs. More importantly, a 3D DNA network structure based on catalytic hairpin assembly was designed for target signal amplification in this work, resulting in an ultralow LOD of 0.33 aM for microRNA-21. In addition, a novel ECL biosensor was developed to detect breast cancer-related genes BRCA1 and BRCA2 simultaneously based on the polarization characteristics of surface plasmon-coupled ECL (SPC-ECL) [75]. In this work, the BNQDs were used as the ECL emitters, and Au NPs and gold-coated silver nanoparticles (Ag@Au NPs) served as the surface plasmon materials. The Au NPs and Ag@Au NPs not only ameliorated the ECL intensity of BNQDs, but also affect the emission polarization mode of QDs due to the SPC effect. As a result, based on the polarization angle-resolved ECL sensor, the BRCA1 and BRCA2 can be detected simultaneously at a single electrode interface.

Introducing heteroatoms into the 2D-QDs is another effective way to improve the performance of electrochemical sensor [76,77]. For example, through a bottom-up synthetic route, the zinc ion-doped MoS_2_ QDs were obtained by Nie and coworkers using ammonium molybdate tetrahydrate and reduced glutathione as the precursors and zinc nitrate hexahydrate as the zinc source [77]. For the fabrication of the ECL system, reduced Cu(I) particles with arm-DNA and capture-DNA (cDNA) were first immobilized on the electrode surface. After introducing pDNA and hybridizing it to the QD-DNA, the system could generate an improved ECL signal in H_2_O_2_, resulting from the attachment of H_2_O_2_ on the sulfur vacancies of Zn-doped MoS_2_ QDs and the coordination with transition metal ions. When target DNA was captured by cDNA, the arm-DNA also bound to QD-DNA. Upon the addition of T7 exonuclease, the arm-DNA was released again as a DNA walker to bind with another QD-DNA. During the cycling of the DNA walker, the Zn-doped MoS_2_ QDs were continuously released from the electrode surface, thereby amplifying the quenching effect of ECL signal. Based on the enhanced ECL intensity of Zn-doped MoS_2_ QDs and reductive Cu(I) particles, the enzyme-assisted DNA walker strategy showed a linear range of HPV 16 DNA from 0.1 to 200 nM with a LOD of 0.03 nM.

### 4.2. 2D-QD-Based Electrochemical Immunological Sensors

Based on the antigen–antibody immunoreactivity, the electrochemical immunological sensor is one of the most common electrochemical methods in the fields of environmental monitoring, medical clinical trials, and protein analysis. Due to the combination of traditional immunoassays with modern electrochemical assays, the electrochemical immunological sensor exhibits both high selectivity and sensitivity. Significantly, the 2D-QDs are able to enhance the performance of immunological sensors by improving the immobilization of biomolecules (e.g., enzymes, antibodies, or DNA) and labels, facilitating the electron transfer, and amplifying the electrochemical signals.

**Figure 3:**
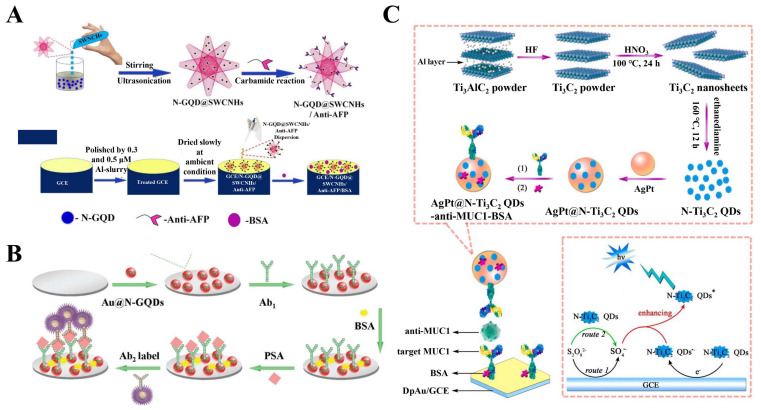
The 2D-QD-based electrochemical immunological sensors. (**A**) Preparation of N-GQD@SWCNHs/anti-AFP bioconjugates and construction of immunological sensors for the detection of AFP. Adapted with permission from Ref. [105]. Copyright 2021 American Chemical Society. (**B**) Construction of the sandwich-type immunological sensor for PSA detection using Au@N-GQDs as the substrate material of electrodes and Au@Ag-Cu_2_O as the labels. Adapted with permission from Ref. [80]. Copyright 2018 Elsevier. (**C**) Synthesis of AgPt@N-Ti_3_C_2_ QDs-anti-MUC1-BSA bioconjugates and fabrication of ECL immunological sensor for the detection of MUC1. Adapted with permission from Ref. [24]. Copyright 2022 Elsevier.

For example, Sun et al. reported a novel electrochemical immunological sensor based on the polyaniline functionalized GQDs (PAGD) for the detection of the heat shock protein 70 (HSP70) [79]. The PAGD composites were prepared using GQDs and aniline as precursors, which were further dropped on the surface of GCE to obtain PAGD/GCE electrode. Compared with GQDs, PAGDs exhibited better electrical conductivity, which can increase the electron transfer rate on the electrode and improve the sensitivity of the biosensor. Then, the PAGD/GCE electrode was incubated with the HSP70 sample to obtain the HSP70/PAGD/GCE basic electrode, which was then incubated with the biotin HSP70 antibody and horseradish peroxidase conjugate streptavidin (HRP-Strept) in sequence. Finally, the electrochemical immunological sensor (HRP-Strept-Biotin-Ab-HSP70/PAGD/GCE) was fabricated. Since HSP70 in the sample can compete with the HRP-labeled HSP70 antibody, the concentration of HSP70 in the sample is negatively correlated with the detection signal of the electrochemical immunological sensor. Under the optimized conditions, HSP70 can be sensitively determined in the range of 0.0976–100 ng/mL with a LOD of 0.05 ng/mL. Recently, an electrochemical immunological sensor was developed by Dutta et al. based on the N-GQDs and single-walled carbon nanohorns (SWCNHs) for the determination of α-fetoprotein (AFP) (Figure 3A) [105]. By assembling N-GQDs and SWCNHs, a hybrid architecture (N-GQD@SWCNHs) was obtained and used to immobilize primary antibodies, anti-AFP. The electrochemical immunological sensor was fabricated by distributing the bioconjugates (N-GQD@SWCNHs/anti-AFP) dispersion on the surface of GCE. Notably, the conductivity and sensitivity of this electrochemical immunological sensor were significantly improved due to the cooperation of N-GQDs and SWCNHs. As a result, a good linear relationship was observed between the cathodic and anodic peak currents and the concentration of AFP from 0.001 to 200 ng mL^−1^ with a LOD of 0.25 pg mL^−1^. In addition, a label-free method for electrochemical ferritin sensing using WS_2_ QDs was reported by Garg and coworkers [45]. In this work, WS_2_ QDs were used for the modification of SPE and as the carriers for the ferritin antibody immobilization. Due to the specific recognition of ferritin antibodies to ferritin, the prepared immunological sensor has high selectivity and reproducibility for the detection of ferritin.

The 2D-QD-based sandwich-type electrochemical immunological sensor is also widely applied in clinical diagnosis and biochemical analysis. For example, Yang et al. developed an ultrasensitive sandwich-type electrochemical immunological sensor (Figure 3B), in which Au@N-GQDs and Au@AgCu_2_O were employed as the substrate material and the label of secondary antibody (Ab_2_), respectively [80]. On the one hand, the Au@N-GQDs NPs can immobilize on the GCE surface through the π–π conjugation effect, which further improve the conductivity of GCE. On the other hand, the Au@Ag-Cu_2_O was not only used as the label of Ab_2_, but it also exhibited good electrocatalytic activity towards the reduction of H_2_O_2_, so as to effectively amplify the current signal for prostate-specific antigen (PSA) detection. This immunological sensor showed an excellent dynamic concentration range from 0.01 pg mL^−1^ to 100 ng^−1^ with a LOD of 0.003 pg mL^−1^. In addition, as the substrate materials to modify electrodes, the 2D-QDs can also employ the emitters, antibody labels, and co-reactants [23,81]. For example, Nie and coworkers developed a novel ECL immunological sensor based on poly(5-formylindole)/reduced graphene oxide nanocomposite (P5FIn/erGO) and AuNP decorated GQDs (GQDs@AuNP) [81]. In this case, the GQDs@AuNP was used to immobilize Ab_2_ and serve as the ECL probe with improved electron transfer capability and stable ECL intensity. Moreover, the electrochemical impedance spectroscopy (EIS) results showed that after the connection of GQDs@AuNP-Ab_2_ to the modified electrode, the diameter of the semicircular domain was significantly reduced owing to the high conductivity of GQDs. The obtained ECL immunological sensor exhibited a wide linear range from 0.1 pg mL^−1^ to 10 ng mL^−1^ and a LOD of 3.78 fg mL^−1^ for the ultrasensitive detection of carcinoembryonic antigen (CEA). Peng et al. prepared a kind of phosphorus and sulfur co-doped GQDs (P, S-GQDs) by electrolysis of a graphite electrode in a solution containing sodium phytate and sodium sulfide [82]. In the presence of K_2_S_2_O_8_, P, S-GQDs exhibited higher ECL activity than GQDs, P-GQDs, and S-GQDs, which can be used as the bright signal indicators after the monoclonal antibody labeling of okadaic acid (anti-OA-MAb). The ECL immunological sensor was fabricated for highly sensitive detection of OA using carboxylated multiwall carbon nanotubes-poly(diallyldimethylammonium) chloride-Au nanocluster composites, modified GCE as the sensing platform and P, S-GQDs as the efficient ECL markers. The determination of OA was achieved in the range 0.01–20 ng mL^−1^ with a low LOD of 5 pg mL^−1^_._ In addition, Jiang et al. constructed an ECL immunological sensor for sensitive determination of mucin 1 (MUC1) based on the nitrogen-doped titanium carbide QDs (N-Ti_3_C_2_ QDs) (Figure 3C) [24]. The N-doped MXene QDs were synthesized via a top-down route by hydrothermal method, which exhibited an enhanced ECL property and possessed higher ECL quantum efficiency than that of Ti_3_C_2_ QDs. For the fabrication of the ECL immunological sensor, the AuNPs-modified electrodes were used to immobilize the MUC1 antibodies. The ECL signaling probe was prepared using AgPt nanospheres to immobilize N-Ti_3_C_2_ QDs, and the MUC1 antibody was linked onto the surface of nanospheres by Ag–N and Pt–N bonds. Moreover, the ECL signal could be further enhanced due to the generation of sulfate radicals. Consequently, the ECL immunological sensor system realized sensitive determination of MUC1 with a low LOD of 0.31 fg mL^−1^.

### 4.3. 2D-QD-Based Electrochemical Enzyme Sensors

Electrochemical enzyme biosensors can present the electron transfer caused by the biochemical reaction between enzymes and analytes as an electrochemical signal for quantitative analysis. The 2D-QDs have stronger affinity for enzymes compared to other 2D materials, such as graphene oxide and reduced graphene oxide [106]. Therefore, the 2D-QDs with large specific surface areas have been widely utilized to immobilize enzymes on electrodes in electrochemical biosensors. In addition, the 2D-QDs as electrode modifiers can accelerate the electron transfer rate and the electrocatalytic activity of enzymatic reactions on the electrode, thereby improving the performance of the biosensor.

**Figure 4:**
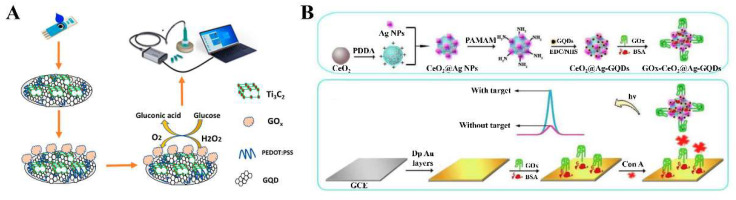
The 2D-QD-based electrochemical enzyme biosensors. (**A**) Fabrication of PEDOT:PSS/Ti_3_C_2_/GQD modified SPCE for glucose detection. Adapted from Ref. [87]. (**B**) Preparation of GOx-CeO_2_@Ag-GQDs and construction of the ECL biosensor. Adapted with permission from Ref. [90]. Copyright 2019 Elsevier.

Due to the advantages of 2D-QDs, such as good conductivity, renewable/reusable electrode surfaces, and low costs, the 2D-QDs can be directly used for enzyme immobilization and electrode modification with simple steps. For example, Baluta and coworkers designed an electrochemical sensing approach for epinephrine detection based on GQDs and laccase modified GCE 85]. For the fabrication of the biosensor, GQDs were firstly spread onto the electrode surface and physically adsorbed for a day to obtain the GQDs/GCE. Then, the laccase was directly dropped onto the modified electrode crosslinked with glutaraldehyde. In addition, the similar procedures can also be used for immobilization of glucose oxidase (GOx) and horseradish peroxidase (HRP) as well as the modification of electrodes [86]. The biggest advantage of this method is simple and cost-effective. However, the sensitivity and stability are issues. Therefore, utilizing the synergistic effect between different 2D-QDs and other materials to further enhance the performance of biosensors is a more preferable strategy. Recently, a label free glucose electrochemical biosensor was developed for the detection of glucose [87]. As shown in Figure 4A, GOx was immobilized on the nanomaterial (PEDOT:PSS/Ti_3_C_2_/GQD)-modified SPCE by a noncovalent interaction strategy. In the presence of glucose, the direct electrochemistry of GOx on the electrode was observed. The presence of Ti_3_C_2_ and GQDs could provide the additional active surface area, resulting in more electroactive sites exposure. Moreover, due to the excellent electrical conductivity and the synergy effect of GQDs, Ti_3_C_2_, and PEDOT:PSS, there was an accelerated electron transfer rate at the electrode/electrolyte interface, which significantly improved the electrochemical behavior of the enzymatic biosensor, achieving a LOD of 65 µM and a high sensitivity of 21.64 µAmM^−1^ cm^−2^. Erkmen et al. reported an amperometric nanobiosensor for the quantitative analysis of phenolic compounds, catechol, epinephrine, and norepinephrine [88]. For electrode modification, GQDs, poly(3,4-ethylenedioxythiophene) nanoparticles (PEDOT NPs) and tyrosinase were dropped onto the surface of a screen printed electrode (SPE) in sequence with 0.25 % glutaraldehyde as the crosslinking agent. As the additional conductive layer provided by GQDs, the SPE/GQDs exhibited stronger current responses and smaller electron transfer resistance compared to the bare SPE. Moreover, due to the superior conductance of PEDOT NPs, the performance of the nanobiosensor was further improved. Under the optimized conditions, the LOD were determined as 0.002, 0.065, and 0.035 μM for catechol, epinephrine, and norepinephrine, respectively. In addition, Salehnia and coworkers prepared a novel nanocomposite (GQD–luminol–AgNP) through a one-step strategy [89]. For the design of the ECL-based biosensor, the nanocomposites and GOx were dropped on the surface of GEC in turn and then dried at room temperature to form the GCE/GQD–luminol–AgNP/GOx. Finally, the Nafion solution was poured on the modified electrode to achieve enzyme immobilization. The increase in ECL signal intensity for luminol could be attributed to the following two aspects: (1) the presence of GQDs increased the specific surface area of electrode; (2) AgNP on the surface of GQDs could provide more active sites with higher electrocatalytic activity. The proposed ECL biosensor displayed the excellent performance towards glucose detection in the concentration range from 25 to 250 mM with a low LOD of 8 mM.

Besides serving as the modifiers of electrodes, the excellent optical properties of 2D-QDs allow them to act as luminophores in ECL sensors. Zuo et al. reported a solid-state ECL biosensor for the detection of Concanavalin A using GQDs as the luminophore [90]. As shown in Figure 4B, the immobilization of GQDs was achieved through the interaction between –NH_2_ of CeO_2_@Ag NPs and –COOH of GQDs, and the nanocomposites were further modified with GOx to obtain the signal probes (GOx-CeO_2_@Ag-GQDs). GOx on the electrode acted as a recognition element to capture Concanavalin A, which further bound with the signal probe to form a sandwich structure. After that, the ground-state GQDs were electrochemically reduced to GQDs^−^, while S_2_O_8_^2−^ was reduced to SO_4_^−^ radicals. Then, the GQDs^−^ and SO_4_^−^ radicals underwent the electron transfer annihilation reaction to generate the excited state GQDs*, which emitted ECL signals when GQDs* fell to the ground state. Both the high loading of GQD luminophore and the good electrical conductivity of Ag NPs were critical to enhance the ECL intensity of GQDs. As a result, the constructed ECL biosensor exhibited the excellent sensitivity to Concanavalin A with a low LOD of 0.16 pg/mL.

### 4.4. 2D-QD-Based Electrochemical Aptasensors

Aptamer is a single-stranded DNA or RNA with high affinity for the targets. Compared with antibodies, aptamers have the advantages of high affinity, low costs, easy synthesis and modification, significant target diversity, and good stability. In electrochemical aptasensors, the immobilization of aptamers on electrodes is a very important part. In recent years, 2D-QDs have been widely used to immobilize aptamers by covalent or non-covalent methods in the construction of an electrochemical aptasensor due to their advantages of large specific surface areas and easy functionalization [97,98]. Moreover, the unique electronic properties of 2D-QDs also allow them to be used as the co-reactants and emitters in ECL biosensors [95,96].

**Figure 5:**
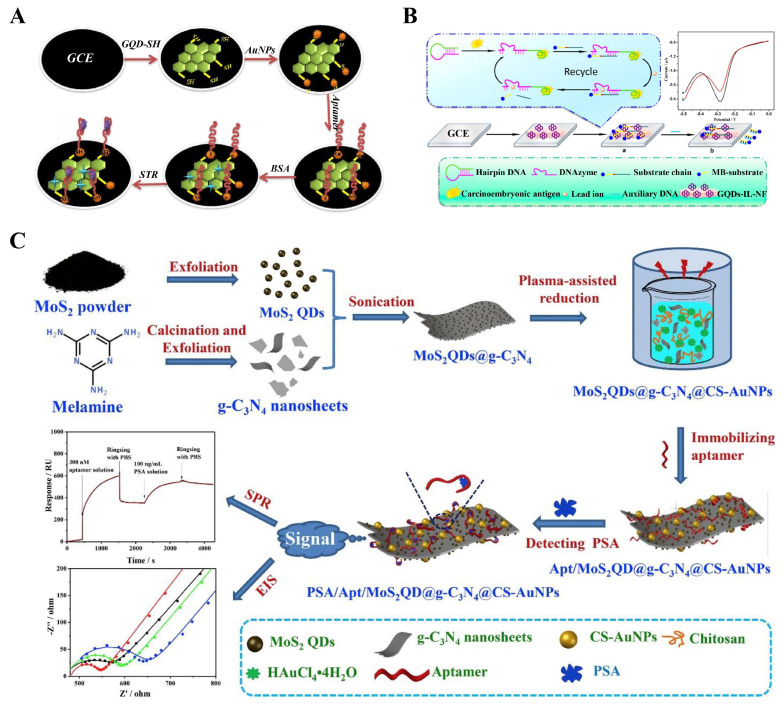
The 2D-QD-based electrochemical aptasensors. (**A**) Construction of electrochemical aptasensor based on AuNPs and GQD-SH for sensitive detection of STR. Adapted with permission from Ref. [91]. Copyright 2018 Elsevier. (**B**) Construction of the electrochemical aptasensor based on GQD-IL-NF nanocomposites for CEA detection combined with Pb^2+^-dependent DNAzyme-assisted signal amplification and the current response before and after the addition of CEA. Adapted with permission from Ref. [93]. Copyright 2018 Elsevier. (**C**) Fabrication of MoS_2_QD@g-C_3_N_4_@CS-AuNPs-based aptasensor for electrochemical detection of PSA and the signal collection using SPR and EIS. Adapted with permission from Ref. [94]. Copyright 2018 Elsevier.

For example, Ghanbari et al. proposed a novel electrochemical aptasensor for highly sensitive detection of streptomycin (STR) based on AuNPs and thiol GQDs (GQD-SH) (Figure 5A) [91]. The AuNPs and GQDs were connected together by Au-S bonds to form the nanocomposites which can be further used to immobilize the aptamers, and the constructed electrochemical aptasensor demonstrated good selectivity and specificity for the determination of STR. However, the stability of this sensor needs to be further improved. Recently, L. Gogola et al. developed a label-free electrochemical aptasensor for the detection of p24-HIV [92]. For the construction of biosensors, GQDs were first immobilized on the SPE by electrodeposition, and the aptamers then linked to the carboxyl group of GQDs via the EDC/NHS reaction. Due to the specific binding of the aptamer to p24-HIV protein, this electrochemical biosensor demonstrated a high selectivity for p24-HIV detection. Khosropour et al. reported an ultrasensitive electrochemical aptasensor for diazinon detection [99]. A new group of nanocomposite (VS_2_ QDs-graphene nanoplatelets/carboxylated multiwalled carbon nanotubes, VS_2_ QDs-GNP/CMWCNTs) was prepared to modify the electrode and immobilize the aptamers. Importantly, all of the VS_2_ QDs, GNP, and CMWCNTs have the ability to increase the surface area and conductivity of electrodes. Due to the synergistic superposition effect of VS_2_ QDs, GNP, and CMWCNTs, the constructed modified electrode showed the excellent conductivity and high electron transfer rate. When the target diazinon specifically bound to the aptamer, the electron transfer on electrode was limited, resulting in the decrease of the DPV peak current and the enhancement of R_CT_ in EIS. As a result, the established electrochemical aptasensor achieved a high sensitivity for quantitative detection of diazinon with a LOD of 11.0 fM and 2.0 fM using DPV and EIS methods, respectively. Huang and coworkers developed an electrochemical aptasensor based on the Pb^2+^-dependent DNAzyme-assisted signal amplification (Figure 5B) [93]. The GQDs-IL-NF composite film composed of Nafion, ionic liquid, and GQDs was used to fabricate the electrode, and meanwhile served as a carrier for DNA immobilization through non-covalent π–π stacking interaction. This aptasensor exhibited greatly enhanced sensitivity due to the signal amplification strategy, which achieved good linearity for CEA detection in the range of 0.5 fg mL^−1^ to 0.5 ng mL^−1^ with a detection limit as low as 0.34 fg mL^−1^.

In recent years, the 2D-QDs, as a class of emerging emitters in the ECL aptasensor system, have received great attention [55,94]. For example, Duan et al. reported a bifunctional aptasensor of SPR and electrochemical techniques for selective and sensitive detection of PSA (Figure 5C) [94]. The 2D g-C_3_N_4_ nanosheets embedded with MoS_2_ QDs and Au NPs were used as the sensitive layers of aptasensor. Thus, the aptamer strands can be immobilized on the surface of nanocomposites through the strong p–p* interaction originating from the g-C_3_N_4_ nanosheets and the high bioaffinity induced by MoS_2_ QDs and Au NPs. In the presence of targets, the aptamer can combine with them to form the aptamer–PSA complex, which can induce an increase in the thickness of the SPR chip or a change in the electrochemical activity of the modified electrode, resulting in a change in SPR or the electrochemical signal. Therefore, the MoS_2_QDs@g-C_3_N_4_@CS-AuNPs nanocomposite was demonstrated as a powerful bifunctional adaptive sensor for the sensitive detection of PSA. In addition, Jiang et al. developed an ultrasensitive all-solid-state ECL platform for kanamycin determination based on a g-C_3_N_4_ QDs/3D graphene hydrogel nanocomposites (CNGH) [100]. For the preparation of CNGH, the g-C_3_N_4_ QDs were first anchored on the GO surface via electrostatic interactions to form g-C_3_N_4_ QD–GO materials, which then self-assembled to form CNGH nanocomposites under the hydrothermal conditions. Then, the CNGH nanocomposites was employed to modify the electrode and immobilize the aptamer. On the one hand, the abundant pore channels in hydrogels can facilitate the mass transport and electron transport in confined spaces, resulting in an enhanced ECL signal emission of g-C_3_N_4_ QDs. On the other hand, the electrochemical activity of CNGH nanocomposites was improved due to the introduction of g-C_3_N_4_ QDs. Therefore, the performance of the ECL platform was significantly improved due to the synergy of g-C_3_N_4_ QDs and graphene hydrogel. As a consequence, the developed ECL sensor achieved the detection of KAN from 1 pM to 50 nM with a LOD of 0.33 pM.

## 5. Summary and Perspectives

The development of 2D nanomaterials is driving the research of 2D-QDs. The 2D-QDs have been successfully prepared by various methods, such as top-down approaches (e.g., ultrasonication-assisted methods, hydro/solvothermal methods, ion intercalation-assisted methods, microwave-assisted methods, and so on) and bottom-up approaches (e.g., hydro/solvothermal methods, external microwave, and laser assisted method, and so on). The 2D-QDs have demonstrated many unique advantages, such as large specific surface areas, easy modification, high electrical conductivity, and good biocompatibility, making them promising candidates in the biological fields. Due to their excellent optical and electrical properties, as well as the high affinity for biomolecules, 2D-QDs have been widely applied in the construction of electrochemical biosensors through serving as the modifiers of electrodes, the carriers of sensitive elements, electron transfer accelerators, luminophores, and so on. Although great progress has been made in the research on 2D-QDs, more efforts are required to further address the challenges as follows. (1) Further understanding of the catalytic, electrochemical, optical, and electrical properties of 2D QDs is needed. (2) The controllable synthesis of 2D-QDs is still quite challenging and new synthetic methods are desired. (3) The research into 2D-QDs has been much less studied than their bulk form. Further, the GQDs dominate the current research on 2D-QDs. The development of 2D-QDs needs more attention and investment. In summary, the current challenges are also opportunities for the development of electrochemical biosensors based on 2D-QDs. For example, the unique properties of ultra-small sizes and large specific surface areas make 2D-QDs promising candidates in the design of wearable electrochemical sensors and devices. In addition, implantable electrochemical biosensors will also be a future research direction. We firmly believe that 2D-QDs will play an important role in future advancements and developments in the fields of chemistry, biomedicine, physiology, and nanomaterials.

## Figures and Tables

**Table 1:** Synthesis of 2D-QDs by top-down methods.

Method	Product Type	Precursors	Size [nm]	Ref.
Ultrasonication assisted method	PGQDs	Natural graphite powder	2–4	[50]
	EGQDs	Expanded graphite powder		
	GOQDs	Graphite oxide powder		
	BPQDs	Black phosphorus	4.9 ± 1.6	[36]
	BPQDs	Black phosphorus	<20	[51]
	g-C_3_N_4_ QDs	Cyanuric acid 2,4-diamino-6-phenyl-1,3,5-triazine	<100	[52]
	g-C_3_N_4_ QDs	Recrystallized dicyandiamide	5–200	[53]
	BNQDs	h-BN	7.71–13.2	[54]
	MoS_2_ QDs	Molybdenum disulfide powder	4.2 ± 0.1	[55]
Hydro/ Solvothermal method	WO_3−x_ QDs	WCl_6_	3.25 ± 0.25	[49]
	N, S-GQDs	Citric acid, thiourea	3.10 ± 0.54	[56]
	N-MXene QDs	Layered Ti_3_C_2_ nanosheet	3.4	[57]
	MoS_2_/WS_2_ dots	MoS_2_/WS_2_ powder	3	[58]
	Ti_3_C_2_ QDs	Ti_3_C_2_ MXene	2.9/3.7/6.2	[59]
	BNQDs	h-BN powder	1.7–10.9	[60]
Ion intercalation-assisted method	MoS_2_ QDs	MoS_2_ powder	3	[61]
	MoS_2_ QDs	MoS_2_ bulk crystal	3.5	[62]
	BN QDs	h-BN flakes	10	[63]
Microwave-assisted method	g-CNQDs	g-C_3_N_4_	3.5 ± 0.5	[64]
	BNQDs	h-BN powder	1.98–7.05	[65]

**Table 2:** Synthesis of 2D-QDs by bottom-up methods.

Method	Product Type	Precursors	Size (nm)	Ref.
Hydro/Solvothermal Method	N-GQDs	Citric acid, urea	4.7±0.5	[66]
	N, S-GQDs	Citric acid, thiourea	4.8 ± 0.5	[67]
External Microwave and Laser Assisted Method	g-CNQDs	Guanidine hydrochloride, EDTA	5	[68]
	S-BN QDs^T^	Boric acid, melamine, thiourea	9.8	[69]
	S-BN QDs^L^	Boric acid, melamine, L-cysteine	9.2	

**Table 3:** Applications of 2D-QDs in electrochemical biosensors.

Type	Sensors	Analyte	LOD	Linear Range	Ref.
DNA Sensors	AuNPs/GQDs/GO/SPCE	microRNA-21	0.04 fM	10^−15^–10^−9^ M	[21]
		microRNA-155	0.33 fM	10^−15^–10^−9^ M	
		microRNA-210	0.28 fM	10^−15^–10^−9^ M	
	H_2_N-GQD/GCE	microRNA-25	0.95 pM	0.3 nM–1.0 μM	[22]
	S-BNQDs/GCE	BRAF	0.3 pM	1 pM–1.5 nM	[69]
	NH_2_-DNA/GQDs/HRP/GE	microRNA-155	0.14 fM	10^−15^–10^−10^ M	[72]
	GQDs/PGE	microRNA-541	0.7 fM	1 fM–1 nM	[73]
	BNQDs/Ru/PtNPs/Nafion/GCE	microRNA-21	0.33 aM	10^−18^–10^−10^ M	[74]
	BNQDs/GCE	BRCA	0.33 fM	10^−16^–10^−9^ M	[75]
	N,S-GQDs@AuNP/GE	DNA	9.4 fM	10^−14^–10^−6^ M	[76]
	Zn-doped MoS_2_ QDs/GCE	HPV 16 DNA	0.03 nM	0.1 nM–0.2 μM	[77]
	BNQDs/BPE	microRNA-141	0.1 aM	10^−^^17^–10^−^^7^ M	[78]
Immunological Sensors	Ab_1_/g-CNQDs/Ag@TCM/GCE	PSA	6.9 fg/mL	10 fg/mL–0.1 pg/mL	[23]
	N-Ti_3_C_2_ QDs/GCE	MUC1	0.31 fg/mL	1 fg/mL–1 ng/mL	[24]
	WS_2_-B QDs/SPE	Ferritin	3.8 ng/mL	10 ng/mL–1.5 μg/mL	[45]
	N,S-GQDs@Au/PANI/Pt	CEA	10 pg/mL	0.5 ng/mL–1 μg/mL	[67]
	HRP-Strept-Biotin-Ab-HSP70/ PAGD/GCE	HSP70	0.05 ng/mL	0.0976–100 ng/mL	[79]
	Au@N-GQDs/GCE	PSA	3 fg/mL	10 pg/mL–0.1 μg/mL	[80]
	GQDs@AuNP-Ab_2_/CEA/BSA/Ab_1_/ AuNP/P5FIn/erGO/GE	CEA	3.78 fg/mL	0.1 pg/mL–10 ng/mL	[81]
	CMCNT-PDDA-AuNC/ GCE	Okadaic acid	0.25 ng/mL	0.01–20 ng/mL	[82]
	AuPdCu/N-GQDs@PS/GCE	HBsAg	3.3 fg/mL	10 fg/mL–50 ng/mL	[83]
	C-TiO_2_@g-CNQDs-Ab_2_/SFN/ Ab_1_/AuNPs/PVPTiO_2_@PFBT/GCE	SFN	0.33 fg/mL	1 fg/mL–100 pg/mL	[84]
Enzyme Sensors	GCE/GQDs/Laccase	Epinephrine	83 nM	1–120 µM	[85]
	GOx-GQD/GCE	Glucose	1.35 µM	10–250 µM	[86]
	PEDOT:PSS/Ti_3_C_2_/GQD/GOx/SPCE	Glucose	65.0 µM	0−500 µM	[87]
	Tyr/GQDs@PEDOT NPs/SPE	Catechol	0.002 μM	0.005–11 μM	[88]
		Epinephrine	0.065 μM	0.2–12 μM	
		norepinephrine	0.035 μM	0.1–2.5 μM	
	Nafion/GOx/GQD–luminol–AgNP/GCE	Glucose	8 μM	25–250 μM	[89]
	GOx-CeO_2_@Ag-GQDs/GCE	Concanavalin A	0.16 pg/mL	0.0005–1.0 ng/mL	[90]
Aptasensors	AuNPs/GQD-SH/GCE	STR	33 fg/mL	0.1 pg/mL–0.7 ng/mL	[91]
	GQDs/SPEs	HIV	51.7 pg/mL	0.93 ng/mL–93 mg/mL	[92]
	GQDs -IL-NF/GCE	CEA	0.34 fg/mL	0.5 fg/mL–0.5 ng mL	[93]
	MoS_2_QDs@g-C_3_N_4_@CS-AuNPs/AE	PSA	0.72 ng/mL	1.0 ng/mL–0.25 ng/mL	[94]
	BSAN/DNA/probe/GE	Lysozyme	29 fg/mL	0.1 pg/mL–0.1 ng/mL	[95]
	Fc-aptamer/BPQDs/RuNDs/GCE	MUC1	6.2 pg/mL	20 pg/mL–10 ng/mL	[96]
	Aptamer/CoPc/NGQDs/GCE	PSA	1.54 pM	34 pg/mL–57 pg/mL	[97]
	GODs@AgNCs@Apt/GE	PGDF-BB	0.82 pg/mL	32.3 fM–1.61 pM	[98]
	VS_2_ QDs-GNP/CMWCNTs/GCE	Diazinon	2.0 fM	10^−14^–1.0–10^−8^ M	[99]
	g-C_3_N_4_ QDs-graphene hydrogel/GCE	Kanamycin	0.33 pM	1 pM–50 nM	[100]

## References

[1] Kim K.K., Hsu A., Jia X., Kim S.M., Shi Y., Hofmann M., Nezich D., Rodriguez-Nieva J.F., Dresselhaus M., Palacios T. (2012). Synthesis of monolayer hexagonal boron nitride on Cu foil using chemical vapor deposition. Nano Lett..

[2] Lee K.H., Shin H.J., Lee J., Lee I.Y., Kim G.H., Choi J.Y., Kim S.W. (2012). Large-scale synthesis of high-quality hexagonal boron nitride nanosheets for large-area graphene electronics. Nano Lett..

[3] Chen P., Li N., Chen X., Ong W.-J., Zhao X. (2017). The rising star of 2D black phosphorus beyond graphene: Synthesis, properties and electronic applications. 2D Mater..

[4] Liu H., Du Y., Deng Y., Ye P.D. (2015). Semiconducting black phosphorus: Synthesis, transport properties and electronic applications. Chem. Soc. Rev..

[5] Kumru B., Antonietti M. (2020). Colloidal properties of the metal-free semiconductor graphitic carbon nitride. Adv. Colloid Interface Sci..

[6] Zhang J., Zhang M., Zhang G., Wang X. (2012). Synthesis of Carbon Nitride Semiconductors in Sulfur Flux for Water Photoredox Catalysis. ACS Catal..

[7] Schwinghammer K., Mesch M.B., Duppel V., Ziegler C., Senker J., Lotsch B.V. (2014). Crystalline Carbon Nitride Nanosheets for Improved Visible-Light Hydrogen Evolution. J. Am. Chem. Soc..

[8] Alhabeb M., Maleski K., Mathis T.S., Sarycheva A., Hatter C.B., Uzun S., Levitt A., Gogotsi Y. (2018). Selective Etching of Silicon from Ti_3_ SiC_2_ (MAX) To Obtain 2D Titanium Carbide (MXene). Angew. Chem. Int. Ed..

[9] Urbankowski P., Anasori B., Makaryan T., Er D., Kota S., Walsh P.L., Zhao M., Shenoy V.B., Barsoum M.W., Gogotsi Y. (2016). Synthesis of two-dimensional titanium nitride Ti_4_N_3_ (MXene). Nanoscale..

[10] Fu Q., Han J., Wang X., Xu P., Yao T., Zhong J., Zhong W., Liu S., Gao T., Zhang Z. (2021). 2D Transition Metal Dichalcogenides: Design, Modulation, and Challenges in Electrocatalysis. Adv. Mater..

[11] Huang J.-K., Pu J., Hsu C.-L., Chiu M.-H., Juang Z.-Y., Chang Y.-H., Chang W.-H., Iwasa Y., Takenobu T., Li L.-J. (2014). Large-Area Synthesis of Highly Crystalline WSe_2_ Monolayers and Device Applications. ACS Nano..

[12] Sun Z., Liao T., Dou Y., Hwang S.M., Park M.-S., Jiang L., Kim J.H., Dou S.X. (2014). Generalized self-assembly of scalable two-dimensional transition metal oxide nanosheets. Nat. Commun..

[13] Xiao X., Song H., Lin S., Zhou Y., Zhan X., Hu Z., Zhang Q., Sun J., Yang B., Li T. (2016). Scalable salt-templated synthesis of two-dimensional transition metal oxides. Nat. Commun..

[14] Liu F., Jang M.-H., Ha H.D., Kim J.-H., Cho Y.-H., Seo T.S. (2013). Facile Synthetic Method for Pristine Graphene Quantum Dots and Graphene Oxide Quantum Dots: Origin of Blue and Green Luminescence. Adv. Mater..

[15] Xu Y., Wang X., Zhang W.L., Lv F., Guo S. (2018). Recent progress in two-dimensional inorganic quantum dots. Chem. Soc. Rev..

[16] Yan Y., Gong J., Chen J., Zeng Z., Huang W., Pu K., Liu J., Chen P. (2019). Recent Advances on Graphene Quantum Dots: From Chemistry and Physics to Applications. Adv. Mater..

[17] Peng D., Zhang L., Li F.-F., Cui W.-R., Liang R.-P., Qiu J.-D. (2018). Facile and Green Approach to the Synthesis of Boron Nitride Quantum Dots for 2,4,6-Trinitrophenol Sensing. ACS Appl. Mater. Interfaces.

[18] Jin Z., Liu C., Liu Z., Han J., Fang Y., Han Y., Niu Y., Wu Y., Sun C., Xu Y. (2020). Rational Design of Hydroxyl-Rich Ti_3_C_2_Tx MXene Quantum Dots for High-Performance Electrochemical N_2_ Reduction. Adv. Energy Mater..

[19] Li D., Liang L., Tang Y., Fu L., Xiao S., Yuan Q. (2019). Direct and single-step sensing of primary ovarian cancers related glycosidases. Chin. Chem. Lett..

[20] Faridbod F., Sanati L.A. (2019). Graphene Quantum Dots in Electrochemical Sensors/Biosensors. Curr. Analy. Chem..

[21] Pothipor C., Jakmunee J., Bamrungsap S., Ounnunkad K. (2021). An electrochemical biosensor for simultaneous detection of breast cancer clinically related microRNAs based on a gold nanoparticles/graphene quantum dots/graphene oxide film. Analyst.

[22] Akbarnia A., Zare H.R. (2018). A voltammetric assay for microRNA-25 based on the use of amino-functionalized graphene quantum dots and ss- and ds-DNAs as gene probes. Mikrochim. Acta.

[23] Liu P., Meng H., Zhang G., Song L., Han Q., Wang C., Fu Y. (2021). Ultrasensitive dual-quenching electrochemiluminescence immunosensor for prostate specific antigen detection based on graphitic carbon nitride quantum dots as an emitter. Mikrochim. Acta.

[24] Jiang X., Wang H., Shen Y., Hu N., Shi W. (2022). Nitrogen-doped Ti_3_C_2_ MXene quantum dots as novel high-efficiency electrochemiluminescent emitters for sensitive mucin 1 detection. Sens. Actuators B Chem..

[25] Li L.-L., Ji J., Fei R., Wang C.-Z., Lu Q., Zhang J.-R., Jiang L.-P., Zhu J.-J. (2012). A Facile Microwave Avenue to Electrochemiluminescent Two-Color Graphene Quantum Dots. Adv. Funct. Mater..

[26] Chung S., Revia R.A., Zhang M. (2021). Graphene Quantum Dots and Their Applications in Bioimaging, Biosensing, and Therapy. Adv. Mater..

[27] Zhou X., Zhang Y., Wang C., Wu X., Yang Y., Zheng B., Wu H., Guo S., Zhang J. (2012). Photo-Fenton Reaction of Graphene Oxide: A New Strategy to Prepare Graphene Quantum Dots for DNA Cleavage. ACS Nano.

[28] Pan D., Zhang J., Li Z., Wu M. (2010). Hydrothermal Route for Cutting Graphene Sheets into Blue-Luminescent Graphene Quantum Dots. Adv. Mater..

[29] Yan X., Cui X., Li L.-s. (2010). Synthesis of Large, Stable Colloidal Graphene Quantum Dots with Tunable Size. J. Am. Chem. Soc..

[30] Zhao H., Chang Y., Liu M., Gao S., Yu H., Quan X. (2013). A universal immunosensing strategy based on regulation of the interaction between graphene and graphene quantum dots. Chem. Commun..

[31] Patnaik S., Martha S., Parida K.M. (2016). An overview of the structural, textural and morphological modulations of g-C3N4 towards photocatalytic hydrogen production. RSC Adv..

[32] Lu Y.-C., Chen J., Wang A.-J., Bao N., Feng J.-J., Wang W., Shao L. (2015). Facile synthesis of oxygen and sulfur co-doped graphitic carbon nitride fluorescent quantum dots and their application for mercury(ii) detection and bioimaging. J. Mater. Chem. B.

[33] Song Z., Li Z., Lin L., Zhang Y., Lin T., Chen L., Cai Z., Lin S., Guo L., Fu F. (2017). Phenyl-doped graphitic carbon nitride: Photoluminescence mechanism and latent fingerprint imaging. Nanoscale.

[34] Garg M., Rani R., Sharma A.L., Singh S. (2021). White graphene quantum dots as electrochemical sensing platform for ferritin. Faraday Discuss..

[35] Angizi S., Alem S.A.A., Hasanzadeh M., Shayeganfar F., Manning M., Hatamie A., Pakdel A., Simchi A. (2021). A Comprehensive Review on Planar Boron Nitride Nanomaterials: From 2D Nanosheets Towards 0D Quantum Dots. Prog. Mater Sci..

[36] Zhang X., Xie H., Liu Z., Tan C., Luo Z., Li H., Lin J., Sun L., Chen W., Xu Z. (2015). Black Phosphorus Quantum Dots. Angew. Chem. Int. Ed..

[37] Gui R., Jin H., Wang Z., Li J. (2018). Black phosphorus quantum dots: Synthesis, properties, functionalized modification and applications. Chem. Soc. Rev..

[38] Gu W., Pei X., Cheng Y., Zhang C., Zhang J., Yan Y., Ding C., Xian Y. (2017). Black Phosphorus Quantum Dots as the Ratiometric Fluorescence Probe for Trace Mercury Ion Detection Based on Inner Filter Effect. ACS Sens..

[39] Xu Z.-L., Lin S., Onofrio N., Zhou L., Shi F., Lu W., Kang K., Zhang Q., Lau S.P. (2018). Exceptional catalytic effects of black phosphorus quantum dots in shuttling-free lithium sulfur batteries. Nat. Commun..

[40] Guo T., Wu Y., Lin Y., Xu X., Lian H., Huang G., Liu J.-Z., Wu X., Yang H.-H. (2018). Black Phosphorus Quantum Dots with Renal Clearance Property for Efficient Photodynamic Therapy. Small.

[41] Liu J., Yi K., Zhang Q., Xu H., Zhang X., He D., Wang F., Xiao X. (2021). Strong Penetration-Induced Effective Photothermal Therapy by Exosome-Mediated Black Phosphorus Quantum Dots. Small.

[42] Sun Z., Xie H., Tang S., Yu X.-F., Guo Z., Shao J., Zhang H., Huang H., Wang H., Chu P.K. (2015). Ultrasmall Black Phosphorus Quantum Dots: Synthesis and Use as Photothermal Agents. Angew. Chem. Int. Ed..

[43] Han G.H., Duong D.L., Keum D.H., Yun S.J., Lee Y.H. (2018). van der Waals Metallic Transition Metal Dichalcogenides. Chem. Rev..

[44] Abid, Sehrawat P., Julien C.M., Islam S.S. (2020). WS_2_ Quantum Dots on e-Textile as a Wearable UV Photodetector: How Well Reduced Graphene Oxide Can Serve as a Carrier Transport Medium?. ACS Appl. Mater. Interfaces.

[45] Garg M., Chatterjee M., Sharma A.L., Singh S. (2020). Label-free approach for electrochemical ferritin sensing using biosurfactant stabilized tungsten disulfide quantum dots. Biosens. Bioelectron..

[46] Xiao S.J., Zhao X.J., Hu P.P., Chu Z.J., Huang C.Z., Zhang L. (2016). Highly Photoluminescent Molybdenum Oxide Quantum Dots: One-Pot Synthesis and Application in 2,4,6-Trinitrotoluene Determination. ACS Appl. Mater. Interfaces.

[47] Xiao S.J., Zhao X.J., Chu Z.J., Xu H., Liu G.Q., Huang C.Z., Zhang L. (2017). New Off-On Sensor for Captopril Sensing Based on Photoluminescent MoO_x_ Quantum Dots. ACS Omega.

[48] Jiang Y., Feng Y., Jiang Y., Liu K. (2019). Improved Current Extraction of Cu/Si Nanowire Heterojunctions for Self-Powered Photodetecting with Insertion of MoO_x_ Quantum Dots Film. ACS Omega.

[49] Wang Y., Wang X., Xu Y., Chen T., Liu M., Niu F., Wei S., Liu J. (2017). Simultaneous Synthesis of WO_3_-_x_Quantum Dots and Bundle-Like Nanowires Using a One-Pot Template-Free Solvothermal Strategy and Their Versatile Applications. Small.

[50] Gao H., Xue C., Guoxin H., Kunxu Z. (2017). Production of graphene quantum dots by ultrasound-assisted exfoliation in supercritical CO_2_/H_2_O medium. Ultrason. Sonochem..

[51] Lee H.U., Park S.Y., Lee S.C., Choi S., Seo S., Kim H., Won J., Choi K., Kang K.S., Park H.G. (2016). Black Phosphorus (BP) Nanodots for Potential Biomedical Applications. Small.

[52] Cui Q., Xu J., Wang X., Li L., Antonietti M., Shalom M. (2016). Phenyl-Modified Carbon Nitride Quantum Dots with Distinct Photoluminescence Behavior. Angew. Chem. Int. Ed..

[53] Liu Q., Zhu D., Guo M., Yu Y., Cao Y. (2019). Facile and efficient fabrication of g-C_3_N_4_ quantum dots for fluorescent analysis of trace copper(II) in environmental samples. Chin. Chem. Lett..

[54] Stengl V., Henych J., Kormunda M. (2014). Self-Assembled BN and BCN Quantum Dots Obtained from High Intensity Ultrasound Exfoliated Nanosheets. Sci. Adv. Mater..

[55] Zhao M., Chen A.Y., Huang D., Chai Y.Q., Zhuo Y., Yuan R. (2017). MoS_2_ Quantum Dots as New Electrochemiluminescence Emitters for Ultrasensitive Bioanalysis of Lipopolysaccharide. Anal. Chem..

[56] Qu D., Zheng M., Du P., Zhou Y., Zhang L., Li D., Tan H., Zhao Z., Xie Z., Sun Z. (2013). Highly luminescent S, N co-doped graphene quantum dots with broad visible absorption bands for visible light photocatalysts. Nanoscale.

[57] Xu Q., Ding L., Wen Y., Yang W., Zhou H., Chen X., Street J., Zhou A., Ong W.-J., Li N. (2018). High photoluminescence quantum yield of 18.7% by using nitrogen-doped Ti_3_C_2_ MXene quantum dots. J. Phys. Chem. C.

[58] Xu S., Li D., Wu P. (2015). One-Pot, Facile, and Versatile Synthesis of Monolayer MoS_2_/WS_2_ Quantum Dots as Bioimaging Probes and Efficient Electrocatalysts for Hydrogen Evolution Reaction. Adv. Funct. Mater..

[59] Xue Q., Zhang H., Zhu M., Pei Z., Li H., Wang Z., Huang Y., Huang Y., Deng Q., Zhou J. (2017). Photoluminescent Ti_3_C_2_ MXene Quantum Dots for Multicolor Cellular Imaging. Adv. Mater..

[60] Liu Q., Hu C., Wang X. (2019). One-pot solvothermal synthesis of water-soluble boron nitride nanosheets and fluorescent boron nitride quantum dots. Mater. Lett..

[61] Qiao W., Yan S., Song X., Zhang X., He X., Zhong W., Du Y. (2015). Luminescent monolayer MoS_2_ quantum dots produced by multi-exfoliation based on lithium intercalation. Appl. Surf. Sci..

[62] Zhou K., Zhang Y., Xia Z., Wei W. (2016). As-prepared MoS_2_quantum dot as a facile fluorescent probe for long-term tracing of live cells. Nanotechnology.

[63] Lin L., Xu Y., Zhang S., Ross I.M., Ong A.C.M., Allwood D.A. (2014). Fabrication and Luminescence of Monolayered Boron Nitride Quantum Dots. Small.

[64] Yin Y., Zhang Y., Gao T., Yao T., Han J., Han Z., Zhang Z., Wu Q., Song B. (2017). One-pot evaporation–condensation strategy for green synthesis of carbon nitride quantum dots: An efficient fluorescent probe for ion detection and bioimaging. Mater. Chem. Phys..

[65] Fan L., Zhou Y., He M., Tong Y., Zhong X., Fang J., Bu X. (2017). Facile microwave approach to controllable boron nitride quantum dots. J. Mater. Sci..

[66] Ganganboina A., Dutta Chowdhury A., Doong R.-a. (2018). N-Doped Graphene Quantum Dots-Decorated V_2_O_5_ Nanosheet for Fluorescence Turn Off-On Detection of Cysteine. ACS Appl. Mater. Interfaces.

[67] Ganganboina A.B., Doong R.A. (2019). Graphene Quantum Dots Decorated Gold-Polyaniline Nanowire for Impedimetric Detection of Carcinoembryonic Antigen. Sci. Rep..

[68] Tang Y., Su Y., Yang N., Zhang L., Lv Y. (2014). Carbon nitride quantum dots: A novel chemiluminescence system for selective detection of free chlorine in water. Anal. Chem..

[69] Liu Y., Wang M., Nie Y., Zhang Q., Ma Q. (2019). Sulfur Regulated Boron Nitride Quantum Dots Electrochemiluminescence with Amplified Surface Plasmon Coupling Strategy for BRAF Gene Detection. Anal. Chem..

[70] Suslick K.S. (1990). Sonochemistry. Science.

[71] Zhu X., Zhang Y., Liu M., Liu Y. (2021). 2D titanium carbide MXenes as emerging optical biosensing platforms. Biosens. Bioelectron..

[72] Hu T., Zhang L., Wen W., Zhang X., Wang S. (2016). Enzyme catalytic amplification of miRNA-155 detection with graphene quantum dot-based electrochemical biosensor. Biosens. Bioelectron..

[73] Akbarnia A., Zare H.R., Moshtaghioun S.M., Benvidi A. (2019). Highly selective sensing and measurement of microRNA-541 based on its sequence-specific digestion by the restriction enzyme Hinf1. Colloids Surf. B Biointerfaces.

[74] Zhang Y., Chai Y., Wang H., Yuan R. (2019). Target-Induced 3D DNA Network Structure as a Novel Signal Amplifier for Ultrasensitive Electrochemiluminescence Detection of MicroRNAs. Anal. Chem..

[75] Liang Z., Nie Y., Zhang X., Wang P., Ma Q. (2021). Multiplex Electrochemiluminescence Polarization Assay Based on the Surface Plasmon Coupling Effect of Au NPs and Ag@Au NPs. Anal. Chem..

[76] Dutta Chowdhury A., Ganganboina A.B., Nasrin F., Takemura K., Doong R.A., Utomo D.I.S., Lee J., Khoris I.M., Park E.Y. (2018). Femtomolar Detection of Dengue Virus DNA with Serotype Identification Ability. Anal. Chem..

[77] Nie Y., Zhang X., Zhang Q., Liang Z., Ma Q., Su X. (2020). A novel high efficient electrochemiluminescence sensor based on reductive Cu(I) particles catalyzed Zn-doped MoS_2_ QDs for HPV 16 DNA determination. Biosens. Bioelectron..

[78] Zhao J., Chen C.X., Zhu J.W., Zong H.L., Hu Y.H., Wang Y.Z. (2022). Ultrasensitive and Visual Electrochemiluminescence Ratiometry Based on a Constant Resistor-Integrated Bipolar Electrode for MicroRNA Detection. Anal. Chem..

[79] Sun B., Wang Y., Li D., Li W., Gou X., Gou Y., Hu F. (2020). Development of a sensitive electrochemical immunosensor using polyaniline functionalized graphene quantum dots for detecting a depression marker. Mater. Sci. Eng. C.

[80] Yang Y., Yan Q., Liu Q., Li Y., Liu H., Wang P., Chen L., Zhang D., Li Y., Dong Y. (2018). An ultrasensitive sandwich-type electrochemical immunosensor based on the signal amplification strategy of echinoidea-shaped Au@Ag-Cu_2_O nanoparticles for prostate specific antigen detection. Biosens. Bioelectron..

[81] Nie G., Wang Y., Tang Y., Zhao D., Guo Q. (2018). A graphene quantum dots based electrochemiluminescence immunosensor for carcinoembryonic antigen detection using poly(5-formylindole)/reduced graphene oxide nanocomposite. Biosens. Bioelectron..

[82] Peng J., Zhao Z., Zheng M., Su B., Chen X., Chen X. (2020). Electrochemical synthesis of phosphorus and sulfur co-doped graphene quantum dots as efficient electrochemiluminescent immunomarkers for monitoring okadaic acid. Sens. Actuators B Chem..

[83] Yan Q., Yang Y., Tan Z., Liu Q., Liu H., Wang P., Chen L., Zhang D., Li Y., Dong Y. (2018). A label-free electrochemical immunosensor based on the novel signal amplification system of AuPdCu ternary nanoparticles functionalized polymer nanospheres. Biosens. Bioelectron..

[84] Huang Y., Zhang S., Lv L., Hong Z., Dai H., Lin Y. (2021). Integrated heterojunction and photothermal effect multiple enhanced ratiometric electrochemiluminescence immunosensor based on calcination controlled and tunable TiO_2_ mesocrystals. Sens. Actuators B Chem..

[85] Baluta S., Lesiak A., Cabaj J. (2018). Graphene Quantum Dots-based Electrochemical Biosensor for Catecholamine Neurotransmitters Detection. Electroanalysis.

[86] Gupta S., Smith T., Banaszak A., Boeckl J. (2018). Graphene Quantum Dots Electrochemistry and Development of Ultrasensitive Enzymatic Glucose Sensor. MRS Adv..

[87] Nashruddin S.N.A., Abdullah J., Mohammad Haniff M.A.S., Mat Zaid M.H., Choon O.P., Mohd Razip Wee M.F. (2021). Label Free Glucose Electrochemical Biosensor Based on Poly(3,4-ethylenedioxy thiophene):Polystyrene Sulfonate/Titanium Carbide/Graphene Quantum Dots. Biosensors.

[88] Erkmen C., Demir Y., Kurbanoglu S., Uslu B. (2021). Multi-Purpose electrochemical tyrosinase nanobiosensor based on poly (3,4 ethylenedioxythiophene) nanoparticles decorated graphene quantum dots: Applications to hormone drugs analyses and inhibition studies. Sens. Actuators B Chem..

[89] Salehnia F., Hosseini M., Ganjali M.R. (2018). Enhanced electrochemiluminescence of luminol by an in situ silver nanoparticle-decorated graphene dot for glucose analysis. Anal. Methods.

[90] Zuo F., Zhang C., Zhang H., Tan X., Chen S., Yuan R. (2019). A solid-state electrochemiluminescence biosensor for Con A detection based on CeO_2_@Ag nanoparticles modified graphene quantum dots as signal probe. Electrochim. Acta.

[91] Ghanbari K., Roushani M. (2018). A novel electrochemical aptasensor for highly sensitive and quantitative detection of the streptomycin antibiotic. Bioelectrochemistry.

[92] Gogola J.L., Martins G., Gevaerd A., Blanes L., Cardoso J., Marchini F.K., Banks C.E., Bergamini M.F., Marcolino-Junior L.H. (2021). Label-free aptasensor for p24-HIV protein detection based on graphene quantum dots as an electrochemical signal amplifier. Anal. Chim. Acta.

[93] Huang J.Y., Zhao L., Lei W., Wen W., Wang Y.J., Bao T., Xiong H.Y., Zhang X.H., Wang S.F. (2018). A high-sensitivity electrochemical aptasensor of carcinoembryonic antigen based on graphene quantum dots-ionic liquid-nafion nanomatrix and DNAzyme-assisted signal amplification strategy. Biosens. Bioelectron..

[94] Duan F., Zhang S., Yang L., Zhang Z., He L., Wang M. (2018). Bifunctional aptasensor based on novel two-dimensional nanocomposite of MoS_2_ quantum dots and g-C_3_N_4_ nanosheets decorated with chitosan-stabilized Au nanoparticles for selectively detecting prostate specific antigen. Anal. Chim. Acta.

[95] Liu H., Zhang Y., Dong Y., Chu X. (2019). Electrogenerated chemiluminescence aptasensor for lysozyme based on copolymer nanospheres encapsulated black phosphorus quantum dots. Talanta.

[96] Yin H., Shi Y., Liu H., Dong Y., Chu X. (2021). Dual-potential electrochemiluminescence of single luminophore for detection of biomarker based on black phosphorus quantum dots as co-reactant. Mikrochim. Acta.

[97] Nxele S.R., Nyokong T. (2021). The effects of the composition and structure of quantum dots combined with cobalt phthalocyanine and an aptamer on the electrochemical detection of prostate specific antigen. Dyes Pigment..

[98] Zhang Z., Guo C., Zhang S., He L., Wang M., Peng D., Tian J., Fang S. (2017). Carbon-based nanocomposites with aptamer-templated silver nanoclusters for the highly sensitive and selective detection of platelet-derived growth factor. Biosens. Bioelectron..

[99] Khosropour H., Rezaei B., Rezaei P., Ensafi A.A. (2020). Ultrasensitive voltammetric and impedimetric aptasensor for diazinon pesticide detection by VS2 quantum dots-graphene nanoplatelets/carboxylated multiwalled carbon nanotubes as a new group nanocomposite for signal enrichment. Anal. Chim. Acta.

[100] Jiang D., Qin M., Zhang L., Shan X., Chen Z. (2021). Ultrasensitive all-solid-state electrochemiluminescence platform for kanamycin detection based on the pore confinement effect of 0D g-C3N4 quantum dots/3D graphene hydrogel. Sens. Actuators B Chem..

[101] Wang Y.-H., Huang K.-J., Wu X. (2017). Recent advances in transition-metal dichalcogenides based electrochemical biosensors: A review. Biosens. Bioelectron..

[102] Song Y., Luo Y., Zhu C., Li H., Du D., Lin Y. (2015). Recent Advances in Electrochemical Biosensors based on Graphene Two-Dimensional Nanomaterials. Biosens. Bioelectron..

[103] Hai X., Li Y., Zhu C., Song W., Cao J., Bi S. (2020). DNA-based label-free electrochemical biosensors: From principles to applications. TrAC Trends Anal. Chem..

[104] Wu L., Erhu X., Zhang X., Xiaohua Z., Chen J. (2014). Nanomaterials as signal amplification elements in DNA-based electrochemical sensing. Nano Today.

[105] Dutta K., De S., Das B., Bera S., Guria B., Ali M.S., Chattopadhyay D. (2021). Development of an Efficient Immunosensing Platform by Exploring Single-Walled Carbon Nanohorns (SWCNHs) and Nitrogen Doped Graphene Quantum Dot (N-GQD) Nanocomposite for Early Detection of Cancer Biomarker. ACS Biomater. Sci. Eng..

[106] Gupta S., Smith T., Banaszak A., Boeckl J. (2017). Graphene Quantum Dots Electrochemistry and Sensitive Electrocatalytic Glucose Sensor Development. Nanomaterials.

